# Polyacrylamide hybridized double networks with polysaccharides and zinc oxide nanoparticles as a Novel approach for removing animal glue stain from paper manuscripts

**DOI:** 10.1038/s41598-026-56366-z

**Published:** 2026-06-15

**Authors:** Hadeer Awad, Mohamed S. Hasanin, Ahmed M. Youssef, Gomaa Abdel-Maksoud

**Affiliations:** 1 Organic Materials Conservation Department, Faculty of Archaeology, Cairo University, P.O. 12613, Giza, Egypt; 2 Cellulose and Paper Department, National Research Centre, Dokki, 12622 Giza Egypt; 3 Packing and Packaging Materials Department, National Research Centre, P.O. 126221, Dokki, Giza Egypt; 4 Center for Converging Sciences and Emerging Technology (CoSET), Benha National University (BNU), Benha, Al Obour 13518, Egypt; 5 Heritage Science Program, School of Humanities, International Business and Humanities, University of Science and Technology (E-JUST), New Borg El-Arab City, 21934 Alexandria Egypt

**Keywords:** Paper manuscript, Animal glue, Polyacrylamide, Polysaccharides, Hybrid double network, Biotechnology, Chemistry, Materials science, Nanoscience and technology

## Abstract

Animal glue stains are commonly found in manuscripts, and their removal is a delicate process that requires selective treatment to avoid damaging the fragile cellulose fibers. Therefore, this study developed a novel hybrid double network hydrogel (HDNH) consisting of synthetic polymer (Polyacrylamide), natural polymers (Agarose or sodium alginate), and Zinc Oxide nanoparticles (ZnONPs) for removing animal glue stains from paper manuscripts. The cleaning mechanism relies on the capillary HDNH to draw the aged animal glue into the gel matrix. To establish the suitability of this HDNH as a paper-cleaning material, the gels were first characterized physicochemically and microstructurally, and the HDNH formulation was confirmed. At the same time, mechanical, physicochemical, morphological, optical, and wettability techniques were performed on the paper samples under investigation, both before and after the cleaning treatment, to evaluate the HDNHs’ cleaning capabilities. The results show that the Polyacrylamide/Agarose/ZnONPs (HDNH/Ag/ZnONPs) provide superior performance in removing most of the accumulated glue layers while maintaining the structural and chemical integrity of the paper substrate. Agarose provides a very rigid, highly porous structure with a superior capacity to retain water, preventing the paper from becoming waterlogged. Polyacrylamide provides flexibility and mechanical durability, allowing the hydrogel to be peeled off in one piece.

**Supplementary Information:**

The online version contains supplementary material available at 10.1038/s41598-026-56366-z.

## Introduction

Paper manuscripts are a popular and important historical and cultural relic. As one of the primary carriers of culture and art, paper plays a vital role in disseminating and transmitting information^1,2^. Initially, paper is structurally rigid; however, its properties deteriorate over time due to various physical, chemical, and biological factors. This degradation is driven by environmental conditions, such as fluctuations in humidity and temperature, air pollution, and dust accumulation^3,4^. Additionally, poor handling, improper storage, inadequate display methods, and the use of traditional adhesives, such as animal glue, are considered the most common deterioration factors of historical paper manuscripts^4–6^.

Animal glue is a highly hygroscopic adhesive derived from animal skins and bones. It exhibits strong adhesion and has extensive applications in various fields^7,8^, such as paper manufacturing, for restoration activities, and as adhesives for bonding and lining prints, drawings, and documents, which were mounted, partly or entirely, on a secondary support by means of glue spots^9,10^. Consequently, yellowing, browning, and embrittlement in the adhesive occur, thus creating distortions, tensions, cockling, and discolouration of documents and graphics^4,9^. Typically, a cleaning process is performed to remove superficial layers that may further degrade the artifact^11,12^. The traditional cleaning procedure may involve immersing paper artwork in water, which can cause the inks to bleed and spread, leading to the distortion of writing over time. It also leads to the swelling of cellulose fibers, which may cause deformation of the paper after drying, resulting in a significant decrease in the mechanical resistance of the cellulosic network^13,14^. Otherwise, the advanced techniques have many limitations such long time of treatment^15^, use harsh solvent effect the paper fibers, and using thermal treatment that aged the fiber as well^16^. The hydrogel cleaning system is considered an essential tool for cleaning ancient books due to its controllability and gentleness^12,17^. Hydrogels can confine cleaning agents within the gel network, achieving the controlled release of the cleaning solution and avoiding the risk of penetration and diffusion of liquid cleaning agents, thereby reducing damage to ancient books^18^, Moreover, they must withstand significant pressure without breaking. At the same time, they must be soft enough to allow the solvent to exert its cleaning action effectively^19^. The swelling behavior of hydrogels enables the effective removal of contaminants without causing unacceptable damage to the underlying paint layers or other delicate materials^20–22^. In this context, the hydrogel materials can be categorized into synthetic hydrogels, such as polyvinyl alcohol and polyacrylamide; natural hydrogels, such as sodium alginate, agarose, chitosan, and cyclodextrin; and hybrid hydrogels that combine two or more hydrogel materials^23–25^.

Acrylamide is a versatile monomer that undergoes polymerization to create polyacrylamide (PAm). The hydrophilic properties of polyacrylamide make it highly useful in conservation due to its excellent water retention capacity and residue-free applications^26–29^. This chemical synergy is crucial for hosting water-based cleaning systems, ensuring the hydrogel remains structurally intact during the treatment of delicate paper substrates while maximizing its sorption efficiency^30,23^.

Natural-based hydrogels, such as polysaccharide-based hydrogels, such as agarose (Ag), were widely used to remove glue from paper and textiles. However, some physical gels have a noncovalent-weak-bonding structure, in which the discontinuous fluid phase (water/solvent) can easily overflow onto surfaces^31, ^^32^,. Additionally, stiff and brittle networks may leave gel residue after cleaning, requiring further water rinsing^33–35^.

Alginate is an unbranched polysaccharide extracted from brown algae. It consists of copolymers of β-D-mannuronic acid and α-L-guluronic acid units linked together by 1,4 glycosidic bonds^36^. The main form of alginate hydrogel is based on ionic cross-linking with multivalent cations, Ba^2+^, Sr^2+^, Ca^2+^, Mg^2+^, and Al^3+^. Due to their interaction with metal cations, as well as their biocompatibility, nontoxicity, and degradability, sodium alginate-based hydrogels have been utilized as adsorbents^37,38^. Combining sodium alginate and acrylamide enables the synthesis of hybrid hydrogels or composite materials that integrate alginate’s environmental friendliness and ion-exchange capabilities with the mechanical robustness and tunable properties of acrylamide-based polymers^26^.

In this context, agarose is a natural polysaccharide derived from marine algae. It is composed of alternating D-galactose and 3,6-anhydro-L-galactopyranose units. It is particularly valued for its thermo-reversible behavior, which allows it to transition between sol and gel, and also has a high capacity to absorb water, is non-toxic before the addition of other cleaning agents, and is stable in both highly alkaline and acidic conditions. This porosity allows the agar gel to act as a molecular sponge^39–41^. Choi et al.^39^, also noted that the significant challenges of agarose are its insufficient mechanical stability, intrinsic hydrophobicity, and, ironically, its thermo-reversible nature. Agarose is relatively hydrophobic compared to other polysaccharides, which makes chemical modification necessary to promote directed intermolecular interactions.

The surface treated with ZnONPs enables precise control of the areas to be cleaned without affecting the rest of the surface^42,43^. ZnONPs possess a broad range of beneficial properties: they are non-toxic, exhibit potent antimicrobial activity, can self-clean surfaces, preventing the buildup of dust or other debris, and are relatively stable chemically and thermally. Due to these properties, they play a crucial role in the development of hydrogels. Also, the incorporation of ZnONPs modified the hydrogel’s internal architecture, creating a more robust framework that enhances its mechanical toughness and peeling stability. This ensures that the gel maintains its integrity during application and removal, preventing any localized failure or residue^44,45^^46^.

Hybrid or nanocomposite hydrogels are made from two or more different molecules obtained from a combination of natural or synthetic materials. The combination of the structure and organization of other molecules within a nanocomposite hydrogel can enhance the hydrogel’s physical, electrical, chemical, and biological properties^30,43,47^. However, current hydrogels still have limitations in practical applications, including poor adaptability to complex surfaces, complex preparation procedures, and the risk of residue formation. Therefore, in this work, to overcome these challenges, optimized polyacrylamide-based hybrid double networks were developed by adding sodium alginate or agarose to the gel network via crosslinking with acrylic acid and acrylamide. Incorporation of ZnONPs further improved the mechanical strength and water absorption capacity. The developed system allows for controlled delivery of the cleaning solution, reducing the penetration of the adhesive, lateral migration of the liquid, and excessive wetting, which can affect the paper fibres. The nanogels can be easily applied and removed without disintegrating or leaving residues. This method offers a mild, efficient and controllable cleaning method for cultural heritage conservation, combining effectiveness with safety and ease of operation.

## Materials and methods

### Materials

Whatman filter paper (pure cotton fibers) was obtained from Sigma–Aldrich (St. Louis, MO, USA), with the following technical information: CAT No. 1442–150, Model quantitative filter Whatman ashless, grade 42, diameter 150 mm. Rabbit skin glue was sourced from Natural Pigments, LLC, U.S. Acrylamide (AM, 99%) was sourced from Sigma –Aldrich, USA. Potassium persulfate (K_2_S_2_O_8_, 99%) and acrylic acid (AAc) were purchased from SD Fine-Chem Limited (SDFCL). India. N, N′-methylene bisacrylamide (MBA, 98%) was purchased from ICSO Research Laboratories Pvt. Ltd. (India), and sodium alginate (SA, chemically pure) was obtained from Shanghai Sinopharm Chemical Reagent Co., Ltd., Shanghai, China. Sodium hydroxide (NaOH), from Piochem, Egypt. Agarose (Ag) was sourced from Vivantis, Malaysia. Zinc oxide nanoparticles (ZnONPs) were sourced from Sigma–Aldrich (St. Louis, MO, USA), fine powders with sizes < 100 nm.

### Preparation of simulated animal glue stains

To simulate the old animal glue layers found in book restoration, a Rabbit Skin solution was prepared by adding 10 g of animal glue granules to 90 mL of deionized water and heating to 60 °C in a water bath with continuous stirring until fully dissolved^4^.

### Preparation of Simulated Stained Paper Samples

The prepared animal glue solution was evenly coated onto a 3 cm × 3 cm filter paper. After coating, the samples were dried at 25 °C in a constant-temperature, humidity-controlled chamber for 24 h to ensure complete curing of the glue layer.

### Accelerated thermal ageing for the stained

The dried stained paper samples were aged for 72 h at 80 °C, at the National Research Centre in Dokki, Giza, Egypt, according to Hassan et al. ^48^.

### Formation of double hydride networks hydrogels (DHNHs)

HDNHs were prepared in three formulae all based on polyacrylamide. In general, a polyacrylamide hydrogel was prepared according to our previous work, with a minor modification^49^. In detail, 5.28 g acrylamide and 0.88 g N, N’ methylene-bisacrylamide were added to 100 mL DW and stirred at 1500 rpm for one h at room temperature. AAc in concentration (1 mol) was added in different ratios (1, 2, and 3 mL), and the pH was adjusted with NaOH. The pH of the reaction process was kept at 7. Potassium persulfate (initiator) was added at a fixed ratio (0.025 g), and the mixture was allowed to polymerize for 30 min at 75 °C in a water bath. The produced hydrogel was observed as a glassy, transparent polymer. In addition, the second formula of the hydrogel was carried out as described above, with the addition of SA at different concentrations (2.5, 5, 10% (w/w)) based on the acrylamide. The third formulation was based on agarose gel in various concentrations (2.5, 5, 10% (w/w)) and acrylamide. Furthermore, ZnONPs were added to the three previous formulations at different ratios (0.5, 1, and 1.5% (w/w)) relative to acrylamide.

### Animal Glue Removal Experiment on Simulated Samples

The hydrogels were cut into 5 mm-thick sheets and placed over the aged animal glue layer, which allowed for partial dissolution of the animal glue and its retention within the polymer matrix. A piece of polyester film and a small acrylic slab were placed on top of the hydrogels. The gel was pressed lightly with the fingers to ensure contact with the animal glue and to keep it in place for 15 min. This time was sufficient to swell and soften the old glue layer without causing excessive moisture to the paper substrate. After 15 min, the gel was lifted off at one corner to visually inspect the consistency of the animal glue. After removing the gel, any remaining glue residue, whether soft or swollen, was gently wiped away using a slightly humid cotton swab. After the hydrogel cleaning process, all samples were placed in a desiccator over anhydrous silica gel for 48 h to reach constant weight. Periodic weighing was performed using an analytical balance (accuracy ± 0.0001 g) until a constant weight was achieved. This step was performed to standardize the moisture content of all samples before morphological and chemical analyses.

### Characterizations

#### ZnONPs Characterisations

The morphology and microstructure of the ZnONPs were examined using a high-resolution transmission electron microscope (JEOL TEM-2100, Japan) operating at 200 kV. To prepare a diluted suspension, the samples were dispersed in ethanol and ultrasonicated. Before imaging, a drop of this suspension was placed on a carbon-coated copper TEM grid. It was then given time to dry completely. The zeta potential of the ZnONPs samples was determined using a Microtrac FLEX particle characterisation system at the Microanalytical Centre, Cairo University, using a quartz cuvette equipped with palladium electrodes. After ultrasonically dispersing the samples in DW, 1 mL of the suspension was added to the cuvette. After equilibration, all measurements were performed at 25.35 °C with an applied current of 0.5 mA.

#### Formulated HDNHs characterizations

The functional groups of the chemical structure of paper samples and hydrogels were analyzed using the Attenuated Total Reflectance - Fourier Transform Infrared Spectroscopy (ATR-FTIR) spectrometer (Thermo Nicolet 6700, Waltham, MA, USA). To investigate the surface morphology of the studied paper samples before and after cleaning with hydrogels, the scanning electron microscope SEM), Quanta 250 FEG 250, equipped with the energy-dispersive X-ray detection system Link Isis 300 (SEM/EDX) at the Egyptian Desalination Research Center of Excellence (EDRC), Egypt, was used.

#### Cleaning evaluations

The cleaning efficiency of different HDNH formulations was evaluated using the weight loss gravimetric method according to ^50^ . Each formulation was tested in three replicates (*n* = 3) to ensure the reproducibility of the results as mentioned with standard deviation (±). Cleaning performance was quantified by absolute weight loss (g). Data were processed, and values for each group were calculated using Eq. (1).

$${\rm \Delta\:W = W_{s} - W_{clean}}$$

where, the initial control sample (W_c_), after staining (W_s_), and after cleaning (W_clean_).

The surface morphology and elemental composition of the paper samples were investigated using Scanning Electron Microscopy (SEM) coupled with Energy Dispersive X-ray Spectroscopy (EDX). Scanning is carried out in non-contact mode. The scanning range is set to 5.00 μm × 5.00 μm. The SEM images were processed using Mountains 11 Software to extract quantitative surface metrics for calculation^51^.

The evaluation of color changes in paper samples, before and after the HDNH treatment, was performed using a Macbeth Color Eye 7000 UV spectrophotometer (USA) at the Conservation Department, Faculty of Archaeology, Cairo University, Giza, Egypt. Color difference **(∆E**) was calculated according to the following Eqs.^52,53^.

$$\:\varDelta\:E=\sqrt{{\left(\varDelta\:\mathrm{L}\right)}^{2}+{\left(\varDelta\:\mathrm{a}\right)}^{2}+{\left(\varDelta\:b\right)}^{2}}$$

where L* defines lightness and varies from 0 (black) to 100 (white)^54^; a* represents the red/green axis, where + a means red and –a means green; b* represents the yellow/blue axis, where + b means yellow and –a means blue. All values of L*, a*, and b* were obtained before treatment and after treatment^55^.

The mechanical properties of the paper samples were tested using a Universal Testing Instrument (DMA-Q 860), measuring strain and stress under static tensile conditions at room temperature (25 °C). Tensile strength and elongation values are reported as mean ± standard deviation, with three independent measurements at a rate of 10 mm/min. The membrane samples were cut to 10.2900 mm × 7.8862 mm × 0.2000 mm and clamped between two automatic gripping units with a gauge length of 3 cm for mechanical loading. The thickness of the membrane samples was measured by an automatic micrometre with a resolution of 1 μm. ATR-FTIR was carried out uing ATR-FTIR spectrometer (Thermo Nicolet 6700, Waltham, MA, USA). The static contact angles of paper samples were measured using the optical tensiometer KRUSS Model DSA. A water drop was placed on the membrane surface with a digital micro syringe, and the contact angle between the water and the membrane was measured when no further change was observed^56^. This test was carried out under environmental conditions for a confidence level of 95%. expanded measurement uncertainty was ± 1 o (coverage factor, k = 2 at a temperature of 22 ± 1 °C and relative humidity of 65 ± 5%.). The expanded measurement uncertainty was ± 1 ^o^ (coverage factor, k = 2 at a temperature of 22 ± 1 for a confidence level of 95%.

The surface topography and roughness of the studied paper samples before and after cleaning with HDNH/Ag/ZnONPs, as the best result, were investigated using a wet-SPM9600 Scanning Probe Microscope (Shimadzu, Japan) at the Atomic Force Lab, Micro Analytical Center, Faculty of Science, Cairo University, Egypt^53^.

## Results and discussion

### ZnONPs Characterizations

**Fig. 1:**
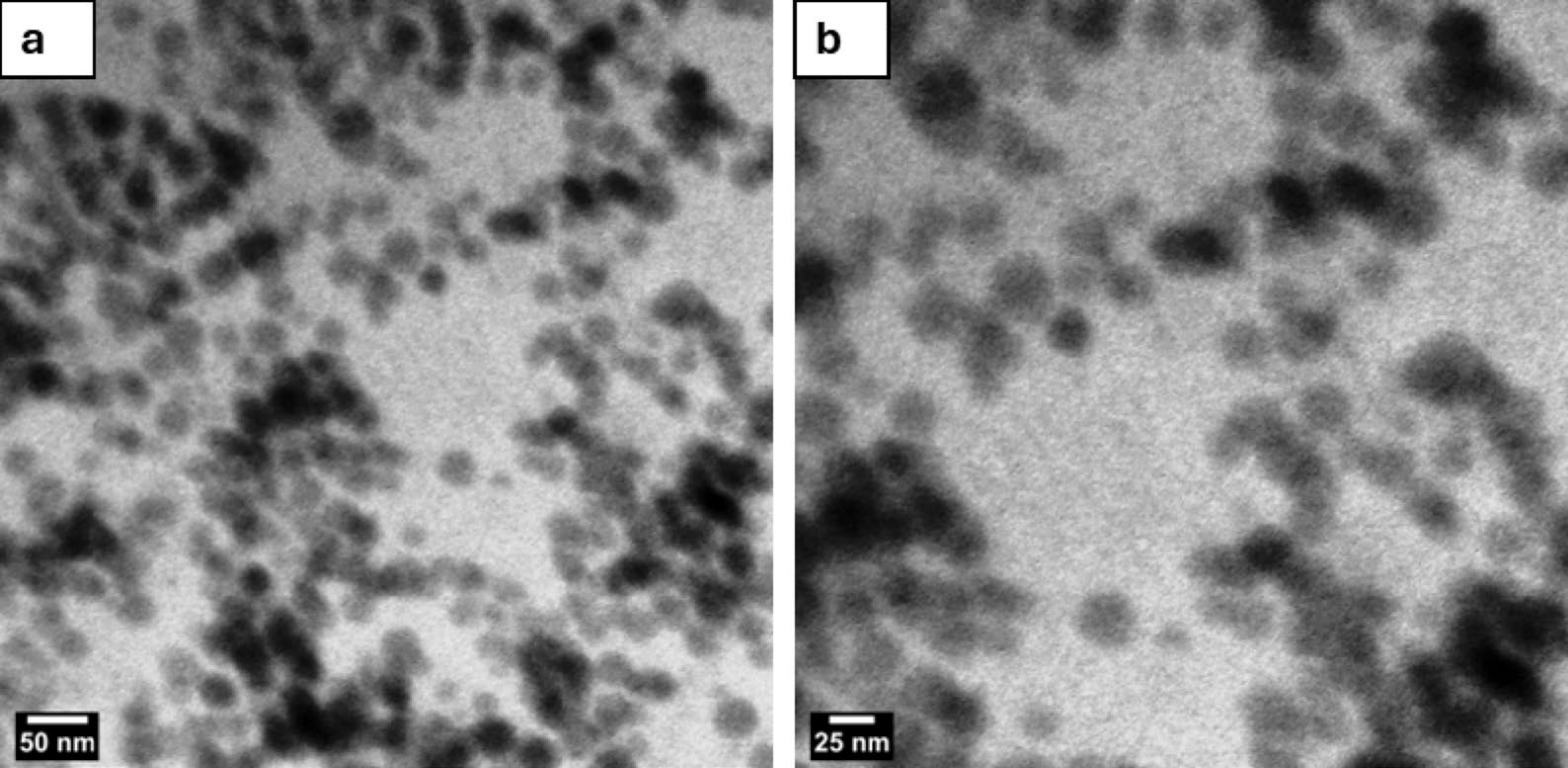
TEM of ZnONPs with low (a) and high (b) magnifications.

The morphology of ZnONPs was examined using TEM images at low and high magnifications, as shown in Fig. 1. TEM images reveal spherical ZnONPs with sharp edges and polydisperse nanoparticles, generally sized between 10 nm and 30 nm. Zeta potential (ZP) measurement was carried out to determine the surface charge and stability of ZnONPs^36,57^. Hence, the surface charge of ZnONPs was determined by zeta potential analysis. ZnONPs exhibited a Zeta Potential of −31 mV, indicating that the nanoparticles are sufficiently charged to generate a strong electrostatic repulsive force, thereby providing good long-term colloidal stability to the suspension. This value also means that the suspension is physically stable and will maintain its monodispersity for a reasonable period.

### Formulated HDNHs optimization

**Fig. 2:**
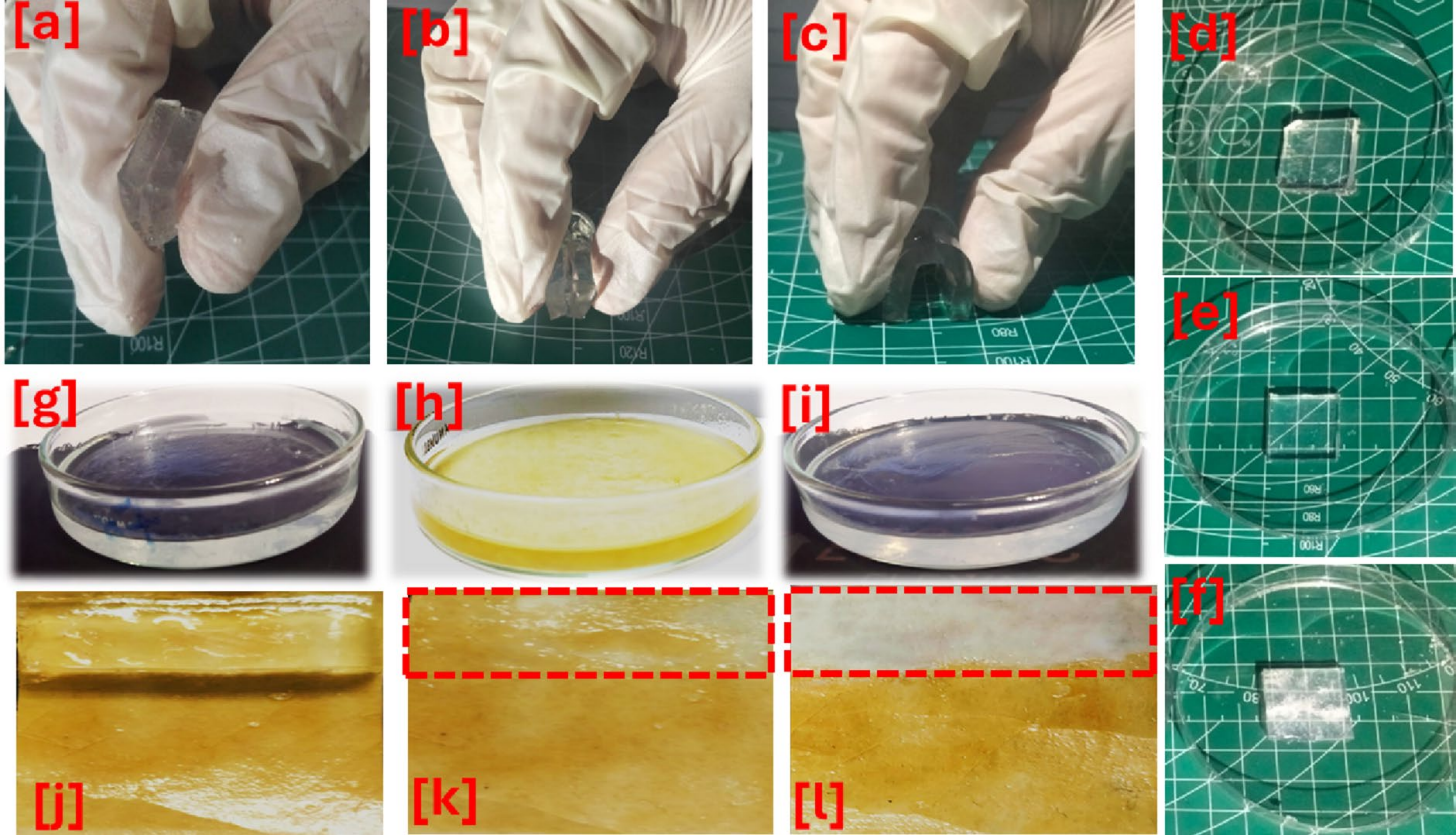
The optimization of the formulation of HDNH: the AAc addition in 1, 2, and 3 mL hydroles digital photo a, b, and c, respectively. The addition of ZnONPs in concentrations 0.5, 1, 1.5%(w/w) digital photo of hydrogel d, e, and f, repsctiviely. The formulated HDNHs formulation, PAm hydrogel (g), HDNH/SA hydrogel (h), HDNH/Ag hydrogel (i). The applying of the HDNH/Ag/ZnONPs on the manuscript (j), manuscript befor cleaning (k) and after cleaning (l).

The formulation of the HDNH was optimized using various factors to produce a suitable hydrogel for cleaning paper manuscripts. Indeed, the HDNH was facilitated by the addition of 1–3 mL of acrylic acid; 1 mL improved its softness and consistency while maintaining the pH at 7. Otherwise, with the increase in AAc to 2 mL, the pH becomes acidic (pH = 6), and the 3 mL gain a pH of 5. The addition of AAc to a polyacrylamide gel (PAm) is typically done to modify the hydrogel’s properties, specifically to introduce carboxylic acid functional groups (-COOH) and subsequently ionize these groups, transforming the neutral PAm into a polyelectrolyte hydrogel known as Poly(acrylamide-co-acrylic acid) (PAm-co-AA), or its partially neutralized form, as shown in Fig. 2. Moreover, the addition of natural polymers was carefully controlled to achieve the desired stickiness and color. For this reason, a concentration range was used to determine the optimal concentration. The ZnONP ratio affects particle dispersity during hydrogel formulation, with a concentration range of 1–1.5%. The 1% concentration was identified as achieving the best dispersity, as shown in Fig. 2. Moreover, the addition of polysaccharides improved the consistency of HDNHs, and HDNH/Ag/ZnONPs were observed to maintain consistency throughout the manuscript cleaning, in comparison to HDNH/SA/ZnONPs. These could be as a result of the sticky nature of SA; however, the two formaldehyde-based, namely, HDNH/Ag/ZnONPs and HDNH/SA/ZnONPs, were the best in handling, respectively. Herein, the thermal impact on this in the case of Ag and the inhibiting impact in the case of the SA played a role in the stability of the formulated structure ^58^.

### Formulated HDNHs characterization

**Fig. 3:**
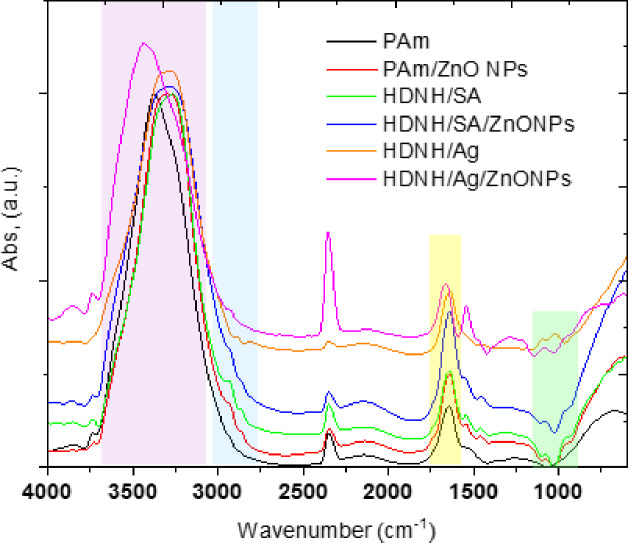
ATR- FTIR spectra of the formulated HDNHs.

The ATR–FTIR spectra of the HDNHs are presented in Fig. 3. All samples exhibit a broad band in the 3300–3500 cm^− 1^ region due to overlapping O-H and N-H stretching vibrations. For neat PAm, this band appears at 3371 cm^− 1^ but shifts to lower wavenumbers in the hybrid systems, indicating stronger intermolecular interactions after the addition of polysaccharides and ZnONPs. The C–H stretching bands are in the range of 2927–2860 cm^− 1^ and show increased intensity in the presence of the polysaccharide components. The characteristic amide I band of PAm at around 1640 cm⁻¹ is observed in all formulations, confirming that the polymer backbone is preserved. The addition of ZnONPs results in slight broadening and small shifts in the O–H/N–H and amide regions, indicating interactions between the surface sites of ZnO and the polymer network’s functional groups. The bands observed around 1600 and 1410 cm^− 1^ in the systems with sodium alginate are attributed to the asymmetric and symmetric stretching of the carboxylate groups (–COO^-^). The bands in the region 1150–1020 cm^− 1^ are associated with C–O–C and C–O vibrations of the polysaccharide structure^59^. The variations in these bands after the addition of ZnONPs suggest interactions of Zn^2^^+^ ions with functional groups of alginates. The spectra in agarose-based systems are characterized by a broad O–H band and features in the 1150–1030 cm^− 1^ region, typical of polysaccharides. The results show that the agarose is incorporated successfully and interacts with the PAm mainly through hydrogen bonding. Slight changes in both high-frequency and fingerprint regions can be seen upon the addition of ZnONPs, indicating the interfacial interactions between ZnO nanoparticles and hydroxyl groups of agarose and amide groups of Pam^60^. The HDNH/Ag spectrum observed the –OH and C–O–C at about (~ 3300–3500 cm^− 1^ (broad band) and ~ 1150–1030 cm^− 1,^ respectively^61^. In sum, the ATR-FTIR results confirm the good incorporation of the different components into the PAm matrix. The observed spectral changes are mainly due to physical interactions, such as hydrogen bonding and coordination, rather than to the formation of new covalent bonds.

### Morphological study

**Fig. 4:**
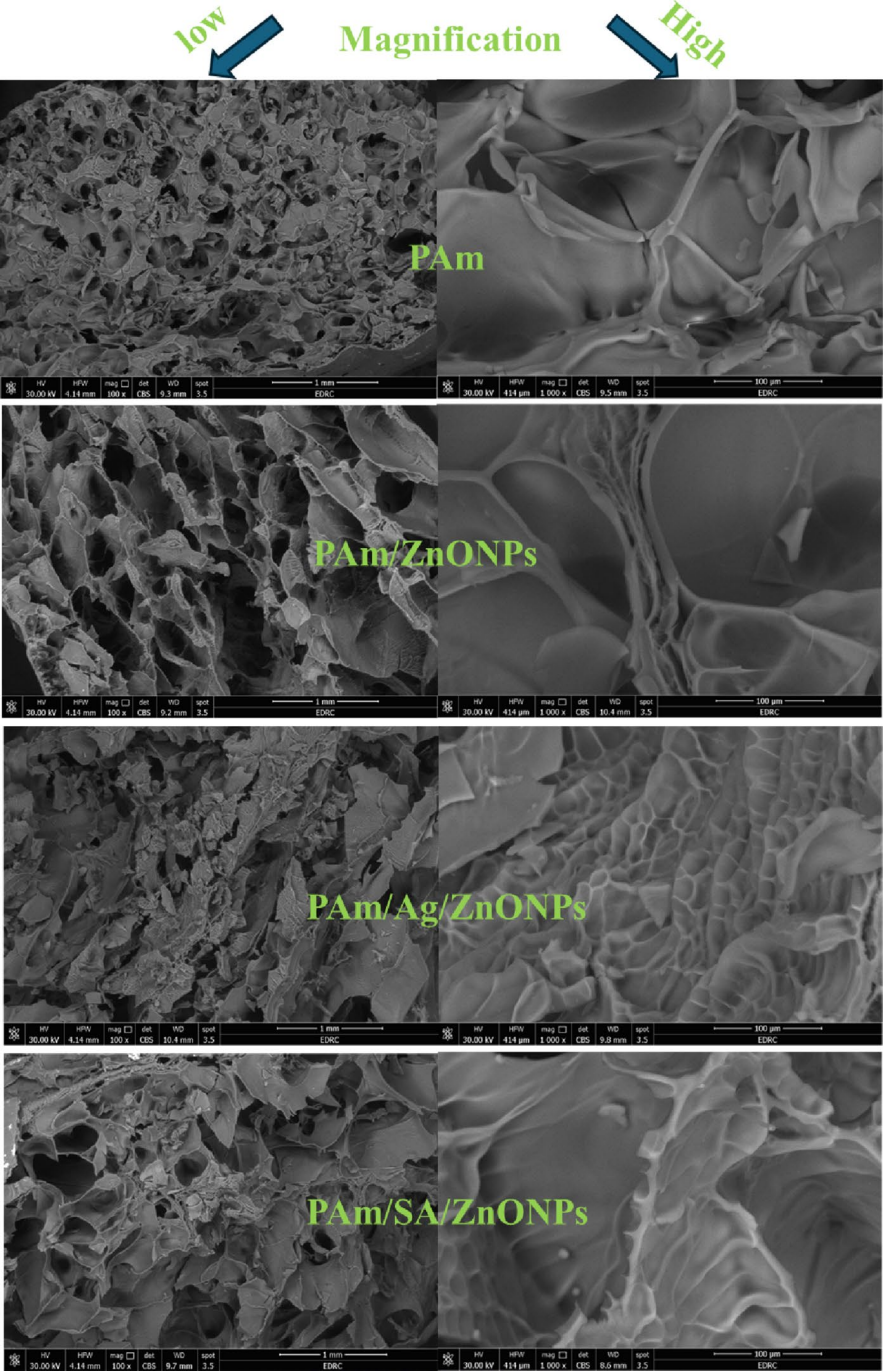
SEM images of formulated HDNH doped with ZnONPs.

Figure 4 illustrates the surface morphology of the formulated HDNHs doped with ZnONPs compared with PAm. A low-magnification PAm SEM image shows a highly porous, sponge-like and multilayer surface structure with irregular size and shape. A high-magnification image of PAm showed smooth, sheet-like pore walls with areas of collapse. The addition of ZnONPs increases both the layer thickness and pore area. The high-magnification image shows that the pores are clearly rounded and expanded, and that the wall between them is strengthened. The HDNH/Ag/ZnONPs appear more compact and denser in low-magnification images than previous formulations. The high-magnification image clearly showed a small pore structure, which could be due to the secondary network formation between the agarose and PAm networks. The SA addition suggests that the HDNHs surface morphology is more tunnel-like in a low-magnification image and appears more folded, with a sheet-like structure. Overall, the morphological study demonstrates that pure PAm displays an irregular sponge-like porosity; the incorporation of ZnONPs alters and strengthens the pore walls, while blending with agarose results in a denser, more microstructured morphology, and blending with sodium alginate creates a highly interconnected, channel-like porous network.

### Cleaned Paper characterization

The cleaning efficiency of the formulated HDNH was evaluated by morphological analysis of cleaned paper using SEM and AFM. Otherwise, the mechanical effect of the cleaning process was studied by assessing the mechanical properties of the documents before and after cleaning, including tensile strength and elongation. The color change was evaluated to affirm the potential of the HDNH as a cleaner.

### Evaluation of Cleaning Efficiency by Weight Loss

**Fig. 5:**
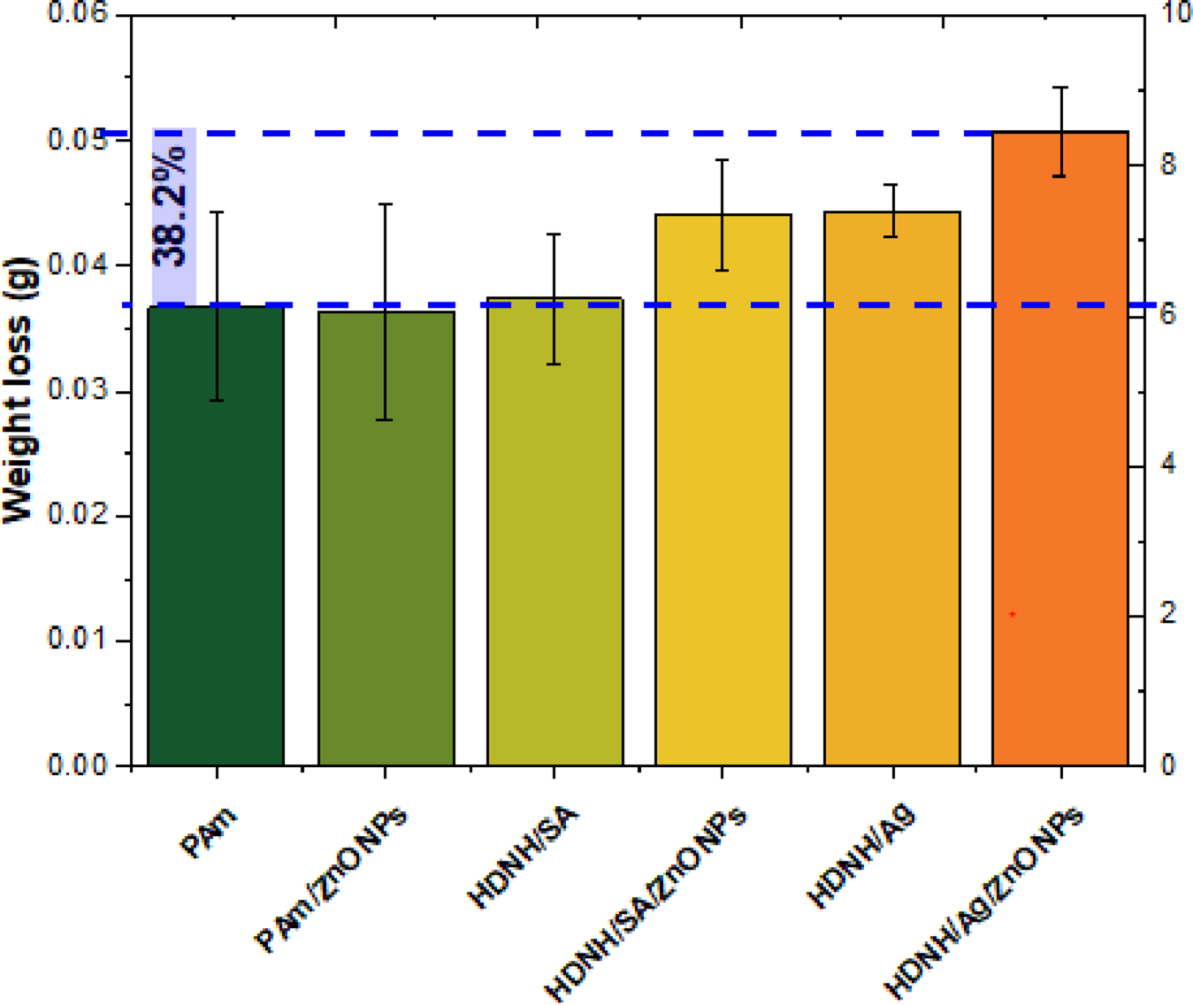
Cleaning efficiency of different hydrogel formulations expressed as weight loss (g).

The results of the gravimetric weight loss of the manuscript after cleaning were presented in Fig. 5. The precision and consistency of each treatment framework allowed for a clear comparison between the basic formulation (PAM) and the improved hybrid gels. The HDNH/Ag/ZnONPs formulation exhibited the highest weight loss of 0.0493 g, offering an enhancement of 38.2% compared with neat PAm. This observation surpasses both pure Pam and the other binary formulations. This significant improvement is attributed to the addition of XnONPs within the polymer matrix. Furthermore, the addition of Agarose transformed the gel into a robust, highly structured network, enhancing its mechanical integrity and surface contact. The low standard deviation observed in these hybrid structures indicates high procedural consistency and reliable performance for surface treatment applications.

Scanning Electron Microscopy equipped with Energy Dispersive X-ray analysis (SEM-EDX).

**Fig. 6:**
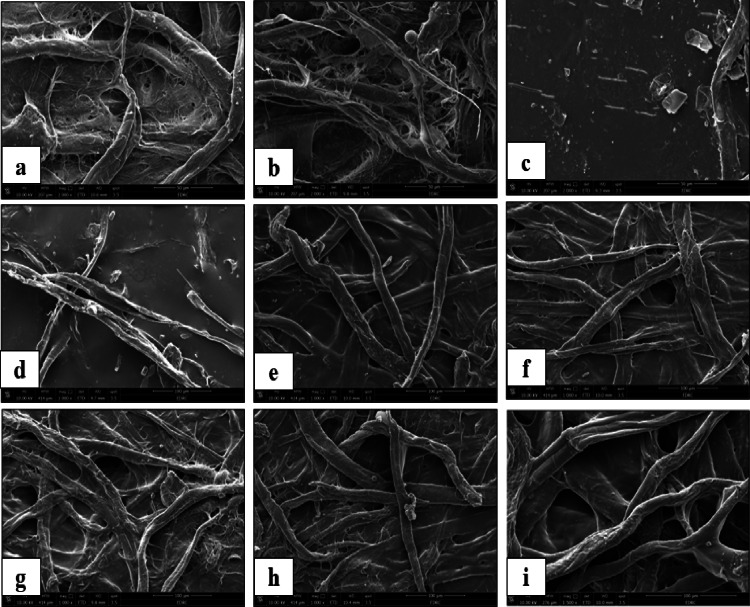
SEM images of cleaned paper samples with acrylamide hybrid: **a**. blank sample, **b**. aged sample, **c**. aged stained sample, **d.** Cleaned sample with PAm, **e**. cleaned sample with PAm/ZnONPs; **f**. cleaned sample with HDNH/SA, **g**. cleaned sample with cleaned sample with HDNH/SA/ZnONPs, **h**. cleaned sample with HDNH/Ag, **i**. and cleaned sample with HDNH/Ag/ZnONPs.

SEM images of cleaned paper samples with acrylamide HDNHs are shown in Fig. 6. The data obtained for the untreated paper sheet (Fig. 6a) showed that a network of intact cellulose fibers existed, with individual fibers and the spaces between them clearly visible. The fibers appear interwoven, as is typical of paper SEM morphology, with a clear fibrous structure. After aging processes (Fig. 6b), accelerated aging was shown to affect the structure, as documented by Hassan & Mohamed^62^. An apparent destruction of paper fibers was observed after aging. Additionally, the roughness of the fibers and the tearing deformation of the paper fibers were observed. After applying animal glue stain to the surface of paper fibers and accelerating aging (Fig. 6c), a thick, smooth, and continuous layer of animal glue is clearly visible, covering the fibers, with small cracks resulting from thermal aging. Schellmann^7^ and Kwan^10^ reported that animal glue stains become noticeably harder, more brittle, and darker with aging. The goal of successful cleaning is to restore the surface to a state as close as possible to the original paper, revealing the cellulose fibers. The use of PAm and PAm/ZnONPs hydrogels on the aged, stained sample resulted in a reasonably effective cleaning process, evidenced by the successful removal of contaminants and the recovery of the paper’s fibrous structure (Fig. 6 d, e). Figure 6 d reveals the presence of residual animal glue, observed as adherent particles within the fiber interstices, which contributes to a marginally less distinct fibrous appearance. Figure 6e shows further improvement, with more exposed fibers because of the removal of a large amount of animal glue stains, but a few residual animal glue stains between fibers were noticed. The results showed that HDNH/SA and HDNH/SA/ZnONPs (Fig. 6f, g) removed a significant portion of the stain. HDNH/Ag and HDNH/Ag/ZnONPs-treated paper, observed in Figs. 6 h and i, respectively. The cellulose fibers are visible and well-defined. The spaces between the fibers are mostly clear, indicating that most of the contaminants have been removed. The best results were obtained with cleaning using HDNH/Ag and HDNH/Ag/ZnONPs, which eliminate the bulk of the glue stains. It should be noted that all the characteristic fibers of the paper surface are clearly visible. It was also observed that gel treatment does not cause damage to paper, i.e., swelling or fraying. These results indicate that cleaning with HDNHs leaves virtually no residue and does not alter the characteristics of paper fibers. The restoration of the paper’s distinctive fibrous appearance after treatment indicates that the gel successfully removed contaminants that had obscured the original paper structure.

To substantiate the findings from SEM microscopy (Table 1), we conducted a thorough digital image processing as shown in Fig. S1. The meteorological capabilities of this powerful software enabled us to quantify several key parameters, including fiber density, cover, and fiber size, as summarized in Figure S1. The image analysis provides clear evidence of cleaning efficiency. The Fiber Coverage percentage, which serves as a proxy for surface exposure and cleanliness, dropped significantly from 47.18% in the blank sample to 28.48% after staining due to the accumulation of animal glue. Following the HDNH/Ag/ZnONPs cleaning treatment with our optimized hybrid hydrogel, the fiber coverage was restored to 45.26%, which is remarkably close to the original control state. Furthermore, the restoration of the Number of Fibers from 981 (stained) back to 1173 (HDNH/Ag/ZnONPs) quantitatively confirms the effective removal of the staining layer and the re-exposure of the underlying fiber network. These metrics strongly correlate with the previously discussed gravimetric weight-loss data (0.0493 g).

**Fig. 7:**
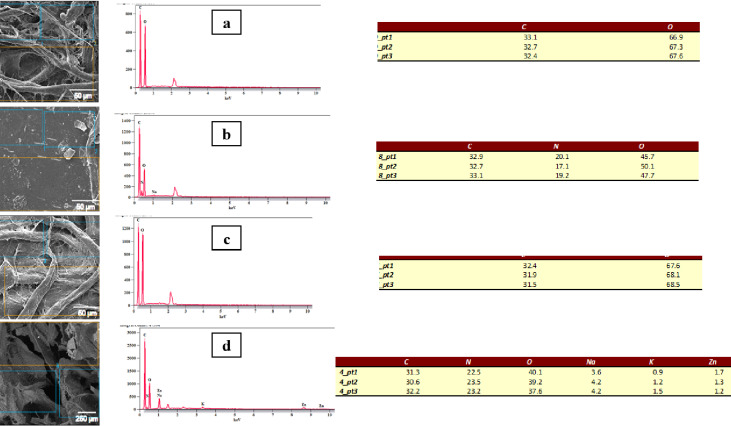
SEM -EDX of cleaned paper samples with acrylamide hybrid: **a**. blank sample, **b**. aged stained sample, **c**. Cleaned sample with HDNH/Ag/ZnONPs, **d**. HDNH/Ag/znONPs.

To assess the absence of cleaning residues and the removal of animal glue stains, EDX analysis was carried out (Fig. 7). The EDX spectrum of stained paper showed a sharp peak for nitrogen (N) at 19.2%. Nitrogen is “chemically marked” as being part of animal glue (protein), which is made up of collagen in aged, stained samples. The elemental composition of the cleaned paper was similar to that of the control sample, with a marked decrease in nitrogen (N), which is part of animal glue. This shows that the HDNH has successfully removed animal glue and paper fibers, as evidenced by the return of carbon and oxygen levels to normal levels, similar to those of the control sample. The EDX spectrum of the original HDNH/Ag/ZnONPs revealed the elemental composition of the system. The presence of zinc (Zn), sodium (Na), and potassium (K) indicates that they are part of the chemicals used in the synthesis of the paper: zinc oxide nanoparticles, sodium hydroxide, and Potassium persulfate (K_2_S_2_O_8_, a catalyst). The fact that there is absolutely no presence of zinc, sodium, and potassium on the cleaned paper, despite their high concentration in the gel matrix, shows that it is the hybrid system is non-migratory and residue-free, thus ensuring the long-term chemical stability of the treated historical paper.

**Table 1:** Quantitative surface measuring of the SEM images processing.

Samples	Mean Fiber Diameter (mm)	Fiber Coverage (%)	Number of Fibers Identified
**Blank paper**	1.092	47.18	1249
**Aged paper**	1.357	43.89	1131
**Aged stained paper**	0.873	28.48	981
**Treated with PAm**	1.619	36.38	630
**Treated with HDNH/Ag/ZnONPs**	1.378	45.26	1173

This result is consistent with the general principles of gel cleaning in conservation, which aim to selectively remove unwanted layers or foreign materials without damaging the substrate^63^. The mechanism of removing animal glue stains from paper manuscript surfaces using acrylamide HDNH depended on the physical processes. Physical swelling: The main mechanism is the slow, vertical dispersion of water from the hydrogel into the animal glue layer. Acrylamide hydrogel retains water within a three-dimensional polymer network. When located on the animal glue, it releases moisture at a controlled rate. Animal glue absorbs this water and undergoes swelling, transitioning from a brittle, hard state to a soft, gel-like state (plasticization). The swelling process generates physical pressure that loosens the strong grip of the dried animal glue on the paper fibers, preparing them for peeling without causing stress or tearing the delicate paper^26,64–66^. In summary, once the glue is softened or partially dissolved, the hydrogel’s capillary network draws the liquefied glue and its degradation products into the gel matrix.

### Change of color

The colorimetric analysis (CIE L∗ a∗ b∗) (Table 2) formulations show that the HDNH formulations successfully restored the optical properties of the paper after animal glue staining and aging. The aging and staining process resulted in a significant decrease in lightness (L*), suggesting minimal darkening from oxidative processes after thermal aging ^67^. However, cleaning with HDNH formulations notably improved the L∗ a∗ b∗ values, indicating the formulations’ effectiveness in restoring the surface’s original color. Among the formulations tested, HDNH/Ag/ZnONPs showed the highest efficiency, with a 17% increase in lightness, a 78% decrease in red-green value (a∗), and a 72% decrease in blue-yellow value (b∗), bringing the substrate close to its original, unstained state.

### Total color difference (ΔE*)

The study evaluated the effectiveness of various hydrogel formulations in removing animal glue stains from paper surfaces by measuring color change. The ΔE value for the aged sample (Table 2) was 1.21, indicating minimal color change. Staining the samples and then thermal aging resulted in significant increases in ΔE of 28.7 and 33.1, respectively, indicating substantial discoloration of the paper surface. After several trials with different gel formulations, a substantial reduction in ΔE values was observed compared to the aged stain sample, demonstrating the gel’s efficiency in removing animal glue stains. HDNH/Ag/ZnONPs showed the smallest color difference (7.95), making it the most successful technique in regaining the original state. This demonstrates that agarose, when combined with acrylamide and ZnO nanoparticles, serves as an effective and compatible medium for removing animal glue from paper surfaces. PAm/ZnONP, and HDNH/SA/ZnONPs showed good results with small color differences (9.32 and 9.19, respectively). HDNH/SA hybrid gel and HDNH/Ag showed moderate efficiency (11.60, 11). PAm hydrogel showed unsatisfactory cleaning results, showing the largest color difference (12.2) from the aged sample. Overall, HDNHs, particularly those based on a ZnO nanoparticle and agarose matrix, are highly advantageous for cleaning contaminated paper artifacts. The improvements noted in ΔE demonstrate their usefulness in paper restoration and conservation. Hassan^59^ stated that the thermal-degradation processes in cellulose include main-chain scission, dihydroxylation, and dehydrogenation, which lead to the formation of several free radicals that cause degradation and yellowing of cellulose. Kwan^10^ reported that animal glue discolors the paper surface and, upon aging, becomes noticeably harder, more brittle, and darker. Cao et al.^68^confirmed that combining SA networks formed after immersion with the existing PAm network yields a double-crosslinked network that can enhance the mechanical properties and adsorption capabilities of the PAm hydrogel.

**Table 2:** The results of color change for the blank sample, aged, stained paper, and treated paper samples.

Before cleaning					
samples	**Color values**				**Total color difference**
	L*	A*	B*		(ΔE*)
Blank sample	92.38	0.82	1.49		---
Aged sample	91.82	0.95	2.55		1.21
Stained samples	71.6	3.08	22,77		28.7
Aged stained sample	70.6	4.07	27.80		33.1

After cleaning (using PAm and PAm/ZnONPs hydrogels)					
Conditions		Color values			Total color difference
		L*	a*	b*	**ΔE**
PAm hydrogel		84.46	0.07	12.18	12.2
PAm/ZnONPs hydrogel		85.96	0.55	9.79	9.32

After cleaning (using HDNH/SA, and HDNH SA/ZnONPs					
conditions		Color values			Total color difference
		L*	a*	b*	**ΔE**
HDNH/SA hybrid gel		84.53	0.27	11.49	11.60
HDNH/SA/ZnONPs		85.88	0.92	9.56	9.19

After cleaning using (HDNH/Ag, and/Ag/ZnONPs					
Conditions		Color values			Total color difference
		L*	a*	b*	**ΔE**
HDNH/Ag		85.95	0.28	11.79	11.00
HDNH/Ag/ZnONPs		85.94	0.52	7.89	7.95

### Mechanical properties

**Fig. 8:**
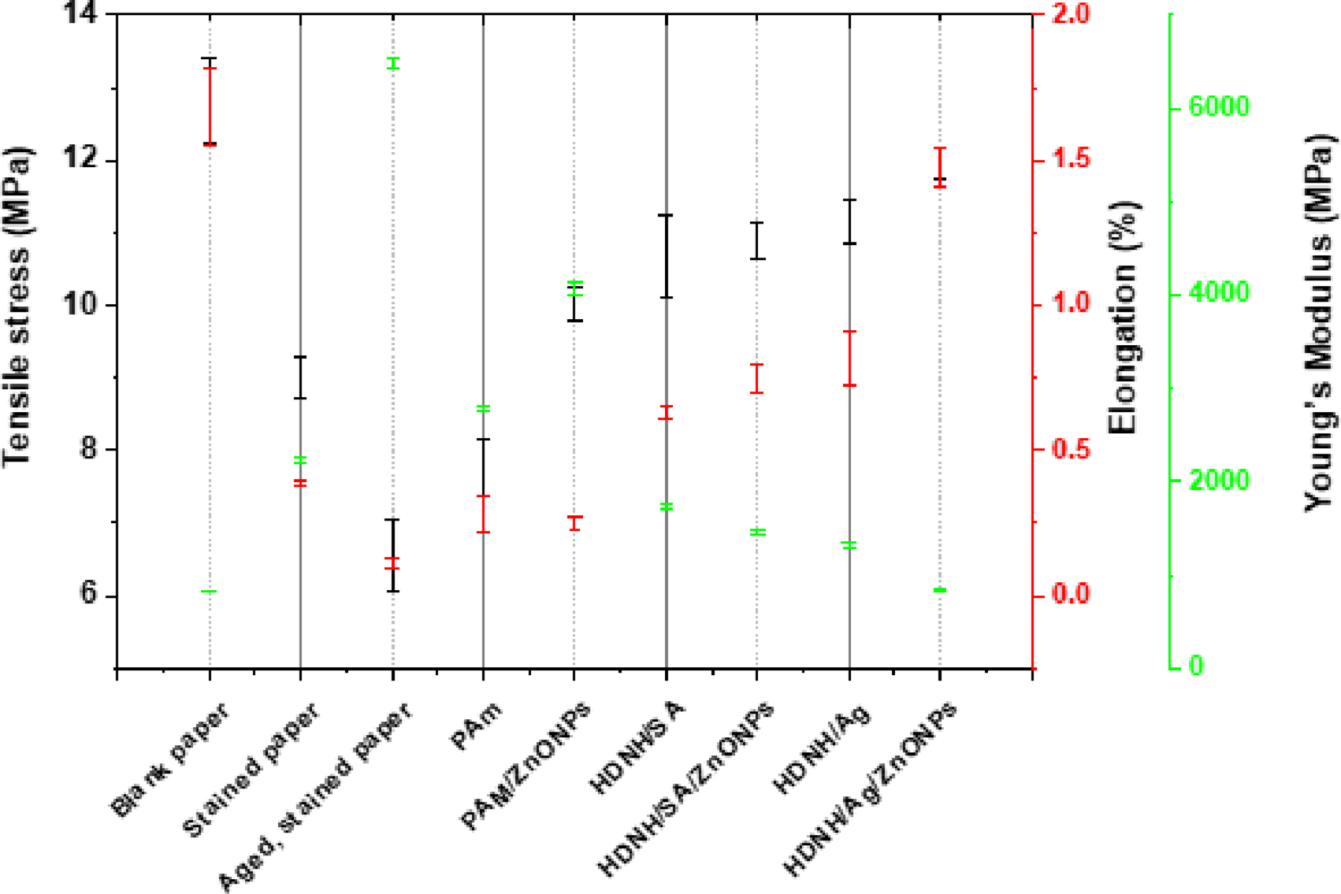
The results of tensile stress and elongation for the blank sample, aged stained paper, and treated paper samples.

The tensile strength (MPa), elongation (%) and Young`s modulus (MPa) of different treated papers are shown in Fig. 8. The values of tensile strength reflect the detailed structure of the paper and the properties of its individual fibers, i.e., the dimensions and strengths of the fibers, their arrangements, and inter-fiber bonding^52^. The aged sample showed a reduction in tensile strength to 6.56 ± 0.49 MPa compared with the blank paper, 12.83 ± 0.58 MPa. Moreover, the elongation decreased from 1.68 ± 0.13 to 0.11 ± 0.01%. It is well known that the inverse proportionality exists between the elongation and Young’s modulus, which increased from 830 ± 7.57 to 6474.6 ± 55.19 MPa for blank and aged paper, respectively. Hassan & Mohamed^62^ It has been confirmed that the effects of accelerated aging processes on paper are explained by cellulose chain splitting, resulting in weaker fibers and the formation of additional hydrogen bonds through covalent bonding, thereby increasing fragility. Schellmann^6^ said that dry conditions cause animal glues to dry out, shrink, and embrittle due to the development of high inner stress and tensile forces within the glue matrix. Herein, the cleaning of the paper using formulated HDNHs affected the mechanical properties behavior. The tensile strength was higher than that of the aged and stained sample, as shown by the fact that the treated samples with HDNH/Ag and HDNH/Ag/ZnONPs observed the highest tensile strengths (11.15 ± 0.31and 11.96 ± 0.21 MPa, respectively) compared to the blank sample. In addition, elongation was recorded as 0.81 ± 0.09% and 1.48 ± 0.06%, with Young’s modules of 1327 ± 25 and 837 ± 17 MPa, respectively. In sum, the mechanical property results confirmed that HDNHs improved the mechanical properties of the cleaning manuscripts, and the HDNH/Ag formulas showed the greatest mechanical improvement. The HDNH/SA formulas improve mechanical properties more than PAm does after the addition of ZnONPs. These observations highlighted the role of ZnONPs in improving mechanical properties.

### Attenuated Total Reflectance Fourier Transform Infrared

**Fig. 9:**
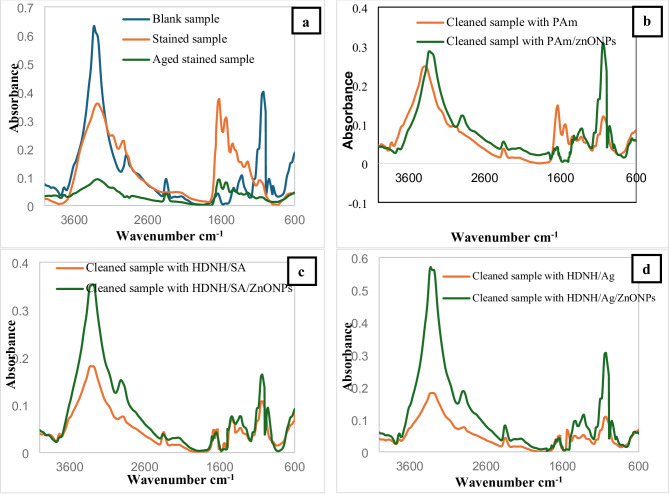
ATR-FTIR of cleaned paper samples with acrylamide HDNH; **a**. blank sample, aged sample, and aged stained sample, **b.** cleaned sample with PAM, and PAm/ZnONPs; **c**.cleaned sample with HDNH/SA, and HDNH/SA/ZnONPs, **d**. cleaned sample with HDNH/Ag, and HDNH/Ag/ZnONPs.

Figure 9 shows the ATR-FTIR spectra of the manuscript with different treatments. The blank (aged) sample in Fig. 9a showed a broadband at 3340.816 cm^− 1^, assigned to (OH-stretching) due to intermolecular hydrogen bonding^2^. It decreased after applying animal glue stain and decreased further after accelerating thermal aging. After cleaning the sample with PAm and PAm/ZnONPs hydrogels (Fig. 9b), some distinctive cellulose functional groups, such as OH and C–H, were observed. Furthermore, the disappearance of specific functional groups indicated the presence of animal glue stains on the paper surface, especially in the PAm-treated samples. After cleaning with PAm and PAm/ZnONPs hydrogels, the O-H intensity increased. The band at 2892.74 cm^− 1^ was assigned to C–H stretching vibrations of the cellulose skeleton, which occur in the region 2700 to 3000 cm^− 1^^69^ which refers to the stretching of phatic groups. After cleaning with PAm and PAm/ZnONPs hydrogels, the C-H intensity increased compared to aged-stained samples. In stained and aged samples with animal glue, the peaks of the animal glue amide I (1646 cm^− 1^) and amide II (1542 cm^− 1^) appeared. Sakr et al., ^70^reported that the bands at 1600 cm^− 1^ (C = O) and 1500 cm^− 1^ (C-N) are characteristic of animal glue, and that the N-H stretching band confirmed the presence of amide A at 3300 cm^− 1^. In stained and aged samples with animal glue, the peaks of the animal glue amide I (1646 cm^− 1^) and amide II (1542 cm^− 1^) appeared^71^]. After cleaning with PAm, the functional group content of animal glue decreased, especially with PAm/ZnONPs hydrogels.

The characteristic peak at 1670 cm^− 1^ corresponds to physically adsorbed water. The band of absorbed and bound water is in the carbonyl region (between 1635 and 1670 cm^− 1^) and can occasionally be rather broad, obscuring the carbonyl bands. For the blank sample, the (C–C) stretching vibration occurs at 1446 cm^− 1^. This band disappeared from the aged, stained sample and reappeared after cleaning with PAm and PAm/ZnONPs hydrogels (Fig. 9b). The bands at 1334 cm^− 1^ were observed in both the blank and stained samples and disappeared after thermal aging. All of these bands (from 1427 cm^− 1^ to 1204 cm^− 1^) are distinct from cellulose fibers and refer to C-O-H, CH_2_ bending vibrations^51^. After cleaning with PAm, PAm/ZnONPs hydrogels appeared. The band (C-O) linked to (OH) appeared at 1041 cm^− 1^ in the blank and stained samples and disappeared after thermal aging. After cleaning with PAm, PAm/ZnONPs, and hydrogels, respectively, the band appeared. The stretching of the (O-C-O) group, which is related to the amount of cellulose present in the blank sample, appeared at a wavelength of 910 cm^− 1^. It was observed that the Reduced C-O Absorbance after staining disappeared after accelerated thermal aging. This indicates that the animal glue layer effectively coats the paper surface, diminishing the spectroscopic signal from the underlying cellulosic fibers. Abdel-Maksoud et al.,^*51*^ said that the bands (from 1159 cm^− 1^ to 1053 cm^− 1^) are distinct from cellulose fibers and refer to various C-O stretching, C-O-C, and C-C-O bending vibrations.

After cleaning, this band was particularly evident in the sample treated with the PAm/ZnONPs hydrogel. It can be said that the disappearance of certain cellulose functional groups and the appearance of certain animal glue functional groups in the sample treated with PAm indicate that some glue stains remained. This suggests that these treatments had a moderate cleaning efficiency. This result confirmed the SEM data, which showed animal glue stains on the treated paper surface and the disappearance of paper fibers in different areas of the examined paper. In contrast, after cleaning with PAm/ZnONPs hydrogels, the characterized functional groups of animal glue almost disappeared. This confirms the superior efficiency of this treatment. From the ATR-FTIR spectra of the treated sample with HDNH/SA and HDNH/SA/ZnONPs (Fig. 9c), the presence of characteristic cellulose peaks at OH (3341 cm^− 1^), CH (2900 cm⁻¹), C = O (1670 cm^− 1^), C-O-H (1349 cm^− 1^), C–O (1049 cm^− 1^) and C–O–C (968 cm^− 1^) related to cellulose. The cleaning efficacy was assessed by monitoring the attenuation of the amide I (1646 cm^− 1^) and amide II (1542 cm^− 1^) absorption bands. The significant reduction in the intensity of these peaks following HDNH/SA treatment, compared to the aged-stained control, indicates successful removal of the animal glue. Similarly, spectra for samples treated with HDNH/Ag and HDNH/Ag/ZnONPs (Fig. 9 d) showed all characteristic cellulose bands, alongside near-total disappearance of amide signals, confirming the superior performance of these formulations in stain removal.

### Contact angle measurement

**Fig. 10:**
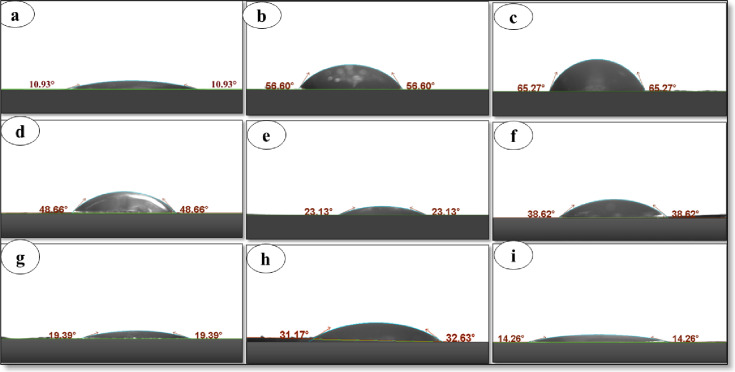
Contact angle of cleaned paper samples with acrylamide HDNH; **a**. blank sample, **b**. aged sample, **c**. aged stained sample, **d.** Cleaned sample with PAM, **e**. cleaned sample with PAm/ZnONPs; **f**. cleaned sample with HDNH/SA,**g**. cleaned sample with cleaned sample with HDNH/SA/ZnONPs, **h**. cleaned sample with HDNH/Ag, **i**. and cleaned sample with HDNH/Ag/ZnONPs.

The contact angle measurement data obtained in Fig. 10 showed the contact angle value (θ) of paper samples before and after cleaning with acrylamide HDNH. In general, the contact angle of the control sample was 10°, indicaing a highly hydrophilic surface. Müller et al.^41^, Abdel-Maksoud et al.^53^, and Barnes et al.^72^, said that hydrophilic surfaces attract water and that a high value of static contact angle indicates that water will be repelled easily at the surface, while a low value < 90 °C shows that water will be retained on the surface.

After applying animal glue to the surface of paper Fig. 10, the contact angle increased to 56.60. Subsequent aging, this value increased to 65.7°. This is due to contaminants such as animal glue, which can drastically alter wetting behavior and improve the contact angle. In the conservation science literature, animal glue layers applied to paper have been shown to reduce water absorption by increasing the contact angle. These adhesive layers can close or clog the paper pores, limiting capillary action and slowing moisture penetration. The adhesive forms a continuous protein layer over the cellulose fibers, thereby reducing the availability of surface hydrophilic functional groups. Furthermore, the presence of hydrophobic amino acid residues and the formation of interconnected protein networks contribute to the material’s resistance to water absorption^67,73,74^and Liu et al.^73^, reported an increase in the adhesive film’s contact angle after aging.

After cleaning the aged stained sample with PAm and PAm/ZnONPs hydrogels (Fig. 10 d, e), the contact angle decreased to 48.66 °, 39.12 °, and 32.13 °, respectively. This indicates the efficiency of PAm in removing some contamination, and we can also observe that the efficiency of acrylamide hydrogels increased by adding ZnONPs. Abdel-Maksoud et al.^53^, demonstrated that the decrease in contact angle is associated with changes in surface chemistry, indicating an increase in surface polarity. This is attributed to the acrylamide gel’s influential role in removing animal glue from the paper surface. Khaksar-Baghant et al.^25^, and Singh et al.^45^,stated that the swelling behavior of acrylamide hydrogels enables effective removal of contaminants without causing unacceptable surface damage. Additionally, ZnONPs-loaded hydrogels exhibit superior moisture retention and swelling behavior, which are crucial for cleaning delicate artifacts without causing damage. After washing with HDNH/SA and HDNH/SA/ZnONPs hybrid (Fig. 10f, g), the contact angle decreased to 38.62° and 19.39°, respectively. This progressive reduction in contact angles shows improved surface wettability and demonstrates the enhanced efficiency of the in removing surface contamination. After cleaning with HDNH/Ag and HDNH/Ag/ZnONPs HDNH is (Fig. 10 h, i), the contact angles decreased to 31.17° and 14.6°, respectively. This decrease in contact angle indicates enhanced surface wettability, reflecting improved removal of animal glue stains. The incorporation of Ag enhanced the cleaning efficiency of the PAm gel, while the addition of ZnONPs further increased this effectiveness.

### **Atomic Force Microscope **(**AFM**)

**Fig. 11:**
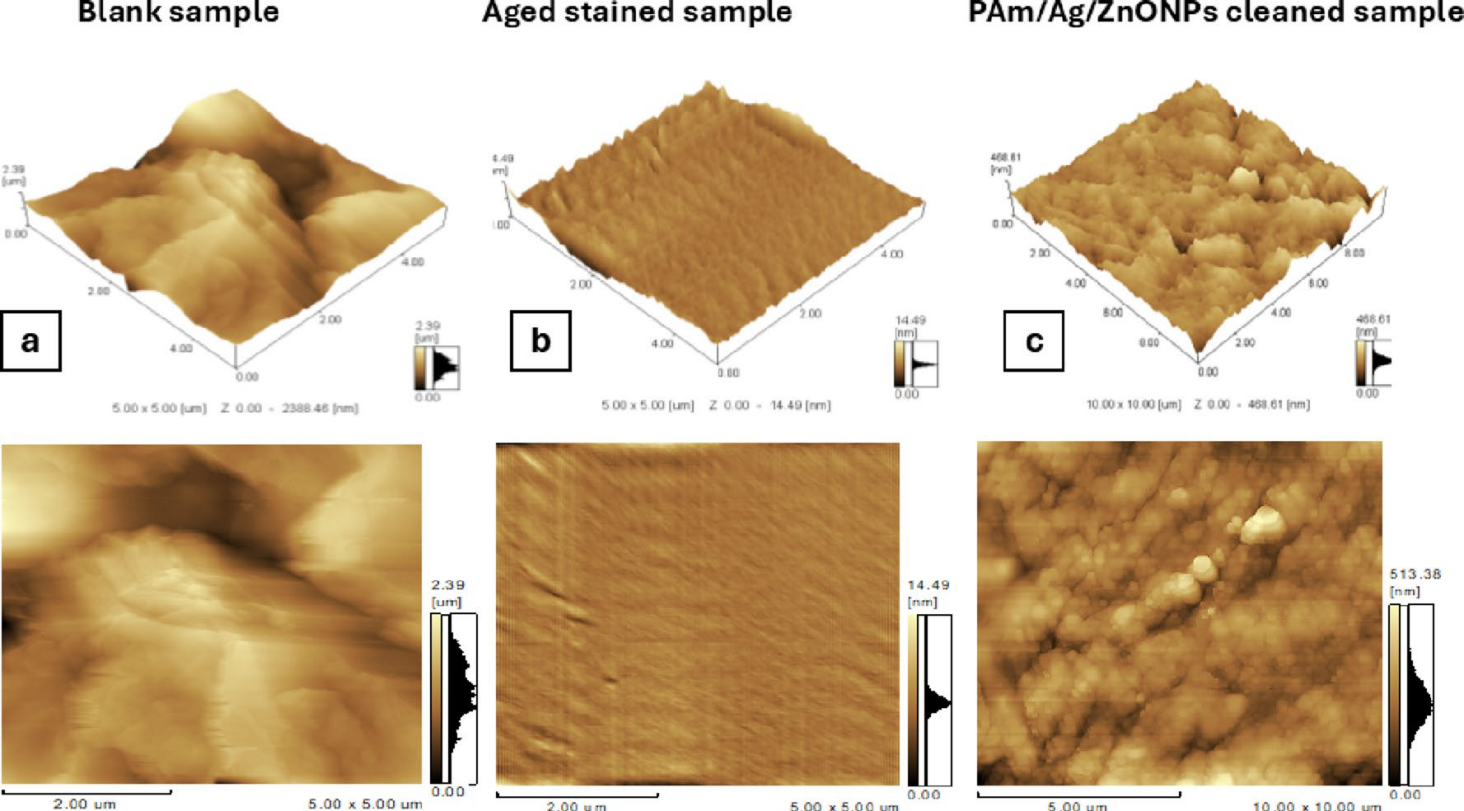
ATR-FTIR of cleaned paper samples with acrylamide HDNH; **a**. blank sample, aged sample, and aged stained sample, **b.** cleaned sample with PAM, and PAm/ZnONPs; **c**.cleaned sample with HDNH/SA, and HDNH/SA/ZnONPs, **d**. cleaned sample with HDNH/Ag, and HDNH/Ag/ZnONPs.

 The AFM images were illustrated in Fig. 11, and measurements were tabulated in Table 3.The surface morphology of paper samples before and after cleaning with HDNH/Ag/ZnONPs is represented in Fig. 11 as the best cleaning system based on previous tests. It was clear from the obtained data (Fig. 11a) that the surface of the control sample was porous, and only portions of the fiber surface appeared. After applying the animal glue stain to the surface and undergoing the accelerated thermal aging process (Fig. 11B), it fills in small gaps, pores, and irregularities between the cellulose fibers, creating a smooth film on the paper surface that covers all the characteristic features of the paper surface and makes the surface less rough. After HDNH cleaning (Fig. 11C), the paper surface was relatively straightforward, resembling the control sample, indicating the removal of the animal glue stain.

The average roughness (Ra) and mean square root (Rq) were measured before and after HDNHs cleaning (Table 3). The results showed that the control sample had roughness Ra of 17.381 nm and Rq of 24.397 nm. After the accumulation of animal glue stains on the paper surface and the application of an accelerated thermal aging process, the roughness Ra and Pq decreased to 0.527nm and 0.759 nm, respectively, due to the accumulation of a smooth layer of animal glue on the paper surface. After cleaning with HDNH/Ag/ZnONPs, the average roughness (Ra) and mean square roughness (Rq) increased and approached those of the blank sample. While complete recovery is often hampered by irreversible structural changes resulting from thermal aging and adhesive penetration, our HDNHs achieved a recovery rate of approximately 73.2%. This indicates that the hydrogel effectively removed the surface layer, and that the cleaning process was gentle enough to avoid excessive abrasion while simultaneously being effective enough to reveal the underlying fiber structure.

Also, as shown by the average values ​​ in Table 3, the sample treated with HDNH/Ag/ZnONPs was highly consistent with the control sample. The mean diameter and mean surface area were normalized to their original levels (0.199 µm and 0.026 µm², respectively) after impurity removal. This statistical consistency implies that the treatment process was not destructive and successfully brought the particles back to their original condition. On the other hand, the stained sample demonstrated a substantial rise in all mean properties. The rise in the mean surface area grew up to (0.041 µm²), and mean Roundness (0.685 µm). This general increase in mean dimensions serves as quantifiable proof of the staining layer’s thickness and successful adsorption. Piantanida et al.^75^, said that AFM imaging allows one to distinguish paper concretely. The presence of materials other than cellulose fibers can be identified using AFM. It is susceptible to degradation and provides new insights into the morphological behavior of cellulose during staining and fiber decomposition^76^. AFM, therefore, has potential applications in the detection of deterioration in library materials and in the added value it provides to preservation research on cultural heritage. Ali^32^ used AFM to evaluate the efficiency of the hydrogel in removing stains from the surface of the artificial and the effect of cleaning methods on surface roughness.

**Table 3:** Mean morphological and surface roughness parameters of surface blank, aged, stained, and cleaned samples using HDNH/Ag/ZnONPs.

**Parameter (Average)**	**Ave rough (Ra)** **(** **nm** **)**	**RMS rough (Rq)** **(** **nm** **)**		**Perimeter** **(μm)**	**Area (μm2)**	**Roundness Index**	**Max Diameter (μm)**
**Control Sample**	17.381	24.397	0.548		0.027	0.78	0.201
**Stained Sample**	0.527	0.759	0.685		0.041	0.68	0.245
**Cleaned sample with** **HDNH** **/Ag/ZnONPs**	12.722	18.157	0.542		0.026	0.79	0.199

## Conclusion

This study evaluated the potential application of PAm hydrogels hybridised with a double network of natural polysaccharides, namely, sodium alginate and agarose, doped with ZnONPs individually, for gently removing animal glue from book surfaces. The formulated hydrogels were characterized physiochemically and topographically, confirming the successful formation of the hybrid hydrogels. On the other hand, the cleaning efficiency of manuscripts was evaluated based on their topographical, physicochemical, and optical properties. Whereas the addition of ZnONPs improved the efficiency of both HDNH removal effects. Moreover, the cleaning process does not require restrictive operating conditions and balances treatment efficacy with safety, ease, and feasibility. Hydrogel can effectively yet gently remove the majority of accumulated glue layers in approximately 15 min, with negligible damage to manuscript fibres and no hydrogel residue, as shown by SEM and EDX. In the case of the HDNH/Ag/ZnONPs HDNH, the characteristic fibrous appearance was restored after treatment, demonstrating the gel’s ability to remove most of the accumulated glue layers that had obscured the paper’s original structure. AFM confirmed that animal glue creates a smooth film that covers all the paper’s characteristic features, making the surface less rough and achieving the lowest ΔE value (7.95) compared with HDNH/SA/ZnONPs (9.19). Additionally, the HDNH/Ag/ZnONPs -treated sample showed the best mechanical properties and the lowest contact angle, indicating high wettability after cleaning. The ATR-FTIR study showed that the spectra of the HDNH/Ag/ZnONPs -treated sample exhibited all characteristic cellulose peaks. The functional groups characteristic of animal glue disappeared. Aged animal glue negatively affects the mechanical properties, leading to reduced ductility due to surface hardening, crack initiation, or contamination. The elongation values for all treated samples with different hydrogel formulations were higher than those of the uncleaned sample. The PAm hydrogel-treated sample yielded the lowest value, indicating that this treatment was ineffective at removing stains from the paper samples. Overall, our results suggest that the HDNH/Ag/ZnONPs can effectively yet gently remove the majority of accumulated glue layers.

## Supplementary Information

Below is the link to the electronic supplementary material.

Supplementary Material 1

## Data Availability

The datasets used and/or analyzed during the current study are available from the corresponding author upon reasonable request.

## Abbreviations

AM: Acrylamide
PAm: Polyacrylamide
HDNH: hybrid double network hydrogel
ZnONPs: Zinc Oxide nanoparticles
Ag: Agarose
SA: Sodium alginate
HDNH/Ag/ZnONPs: Polyacrylamide/Agarose/ZnONPs
HDNH/SA/ZnONPs: Polyacrylamide/ sodium alginate/ZnONPs
AAc: acrylic acid
MBA: N, N-methylene bisacrylamide
NaOH: Sodium hydroxide
EDRC: Egyptian Desalination Research Center of Excellence
ATR-FTIR: Attenuated Total Reflectance - Fourier Transform Infrared Spectroscopy
**(∆E**): Color difference
ZP: Zeta potential
W_c_: control sample
W_s_: after staining
W_clean_: after cleaning
ΔW: weight loss
TEM: Transmission electron microscope
SEM: Scanning electron microscope
SEM-EDX: Scanning Electron Microscopy equipped with Energy Dispersive X-ray analysis
AFM: Atomic force microscope
Ra: Average rough
Rq (RMS): Root-mean-square rough
