# Supplementary material for: Polyacrylamide hybridized double networks with polysaccharides and zinc oxide nanoparticles as a Novel approach for removing animal glue stain from paper manuscripts
Source: Sci Rep. 2026 Jun 15;16:18522. doi: 10.1038/s41598-026-56366-z (PMC13270106; doi:10.1038/s41598-026-56366-z)
Supplement: Supplementary file 1 — Supplementary Material 1 [file 41598_2026_56366_MOESM1_ESM.docx]

**Polyacrylamide hybridized double networks with polysaccharides and zinc oxide nanoparticles as a Novel approach for removing animal glue stain from paper manuscripts**

**Hadeer Awad^1*^, Mohamed S. Hasanin^2*^, Ahmed M. Youssef^3, 4^, Gomaa Abdel-Maksoud^5,1^**

1. Organic Materials Conservation Department, Faculty of Archaeology, Cairo University, P.O. 12613, Giza, Egypt.
2. Cellulose and Paper Department, National Research Centre, Dokki, Giza 12622, Egypt.
3. Packing and Packaging Materials Department, National Research Centre**,** P.O. 126221, Dokki, Giza, Egypt.
4. Research, Development and Innovation Center for Converging Sciences and Emerging Technologies (RDI CoSET Center), Benha National University, El-Obour City, P.O. 11828, Egypt
5. Heritage Science Program, School of Humanities, International Business and Humanities, Egypt-Japan University of Science and Technology (E-JUST), New Borg El-Arab City, 21934, Alexandria, Egypt.

Corresponding Authors: Mohamed S. Hasanin ([sido_sci@yahoo.com](mailto:sido_sci@yahoo.com)), Hadeer Awad ([hadeerawad@cu.edu.eg](mailto:hadeerawad@cu.edu.eg)).

| **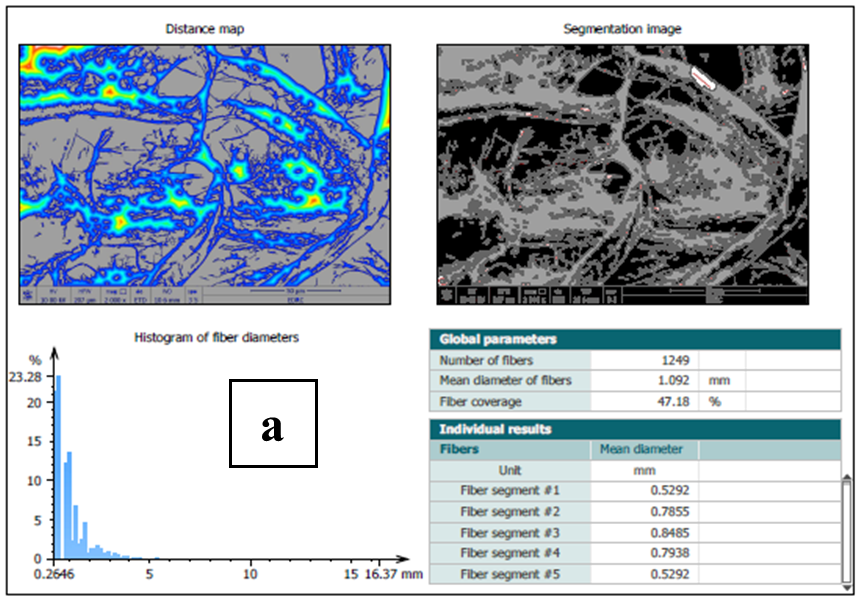** |
| --- |
| **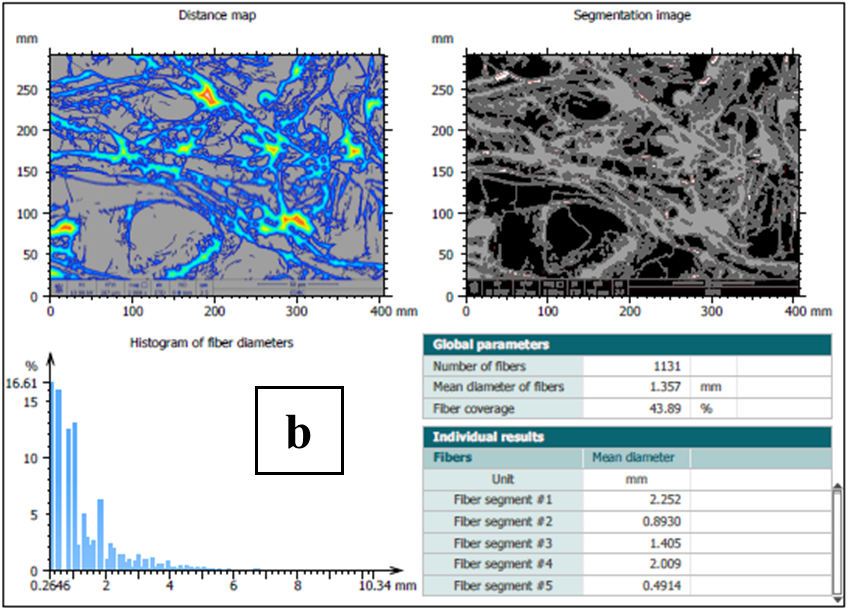** |
| **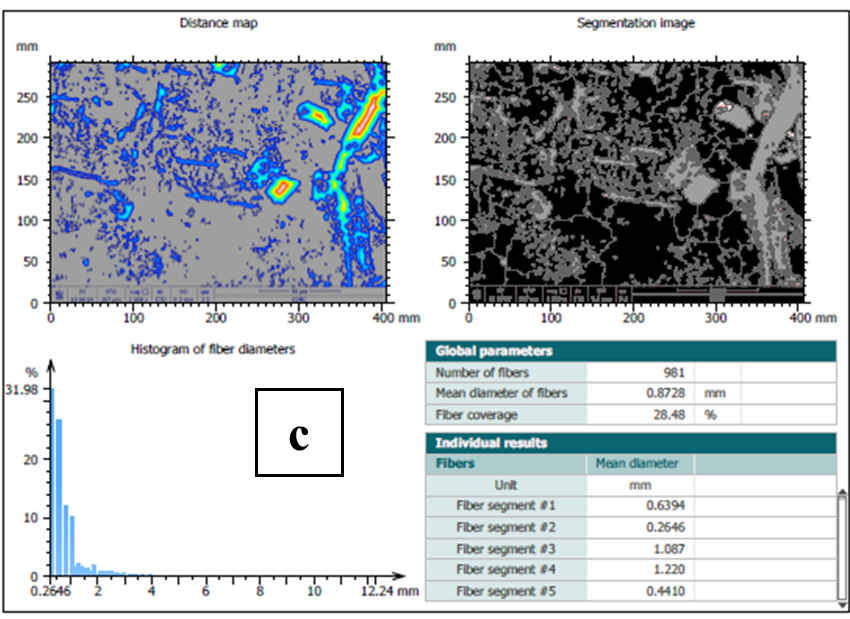** |
| **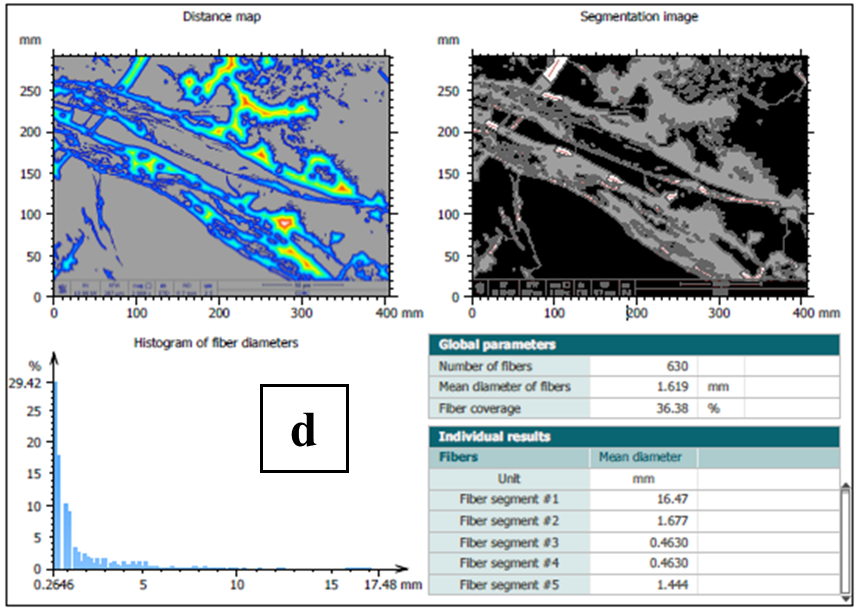** |
| **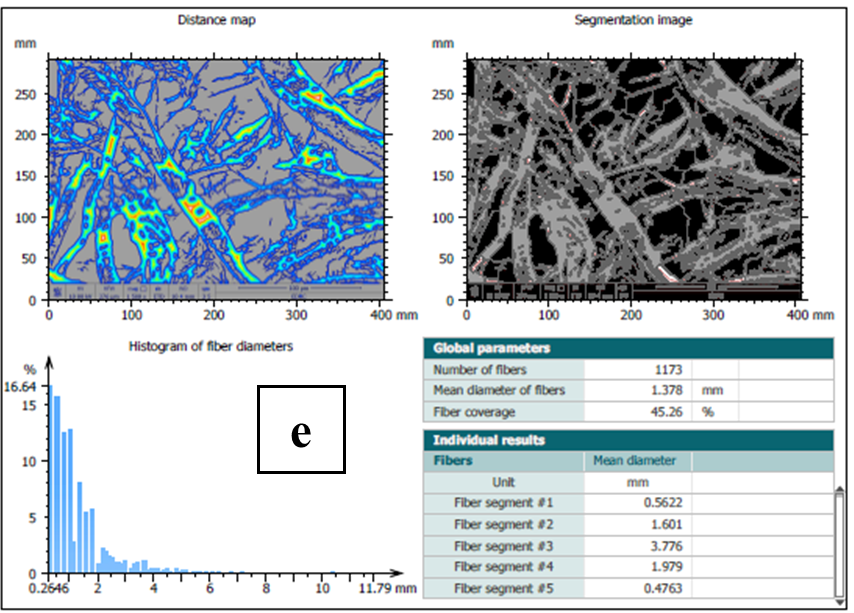** |

**Fig s1**: Quantitative Surface Metrics Extracted via Mountains® 11 Software cleaned paper samples with acrylamide hybrid: a. blank sample, b. aged sample, c. aged stained sample, d. Cleaned sample with PAM, e. cleaned sample with HDNH/Ag/ZnONPs.
